# Stress dependence of indentation modulus for carbon fiber in polymer composite

**DOI:** 10.1080/14686996.2019.1600202

**Published:** 2019-04-26

**Authors:** Hongxin Wang, Han Zhang, Daiming Tang, Kenta Goto, Ikumu Watanabe, Hideaki Kitazawa, Masamichi Kawai, Hiroaki Mamiya, Daisuke Fujita

**Affiliations:** a Research Center for Advanced Measurement and Characterization, National Institute for Materials Science, Tsukuba, Japan; b International Center for Materials Nanoarchitectonics, National Institute for Materials Science, Tsukuba, Japan; c Research Center for Structural Material, National Institute for Materials Science, Tsukuba, Japan; d Systems and Information Engineering, University of Tsukuba, Tsukuba, Japan

**Keywords:** Atomic force microscopy, indentation, 10 Engineering and Structural materials, 104 Carbon and related materials, 500 Characterization

## Abstract

Elastic modulus measured through atomic force microscopy (AFM)-based indentation on single carbon fiber (CF) is found with dependence on lateral applied stress. An *in situ* indentation experiment inside a high-resolution transmission electron microscope was performed to quantitatively understand this phenomenon by observing microstructure change in the indented area. Change of graphitic basal plane misalignment angle during indentation was linked to a continuous change of modulus with the help of finite element simulation. The established relationship between modulus and indentation force was further used to calculate residual stress distribution in CF imbedded in a CF reinforced polymer composite using the AFM indentation technique. The stress-induced formation of nanoscale defects in the CF and their transformation into fracture were directly characterized.

## GRAPHICAL ABSTRACT

**Figure:**
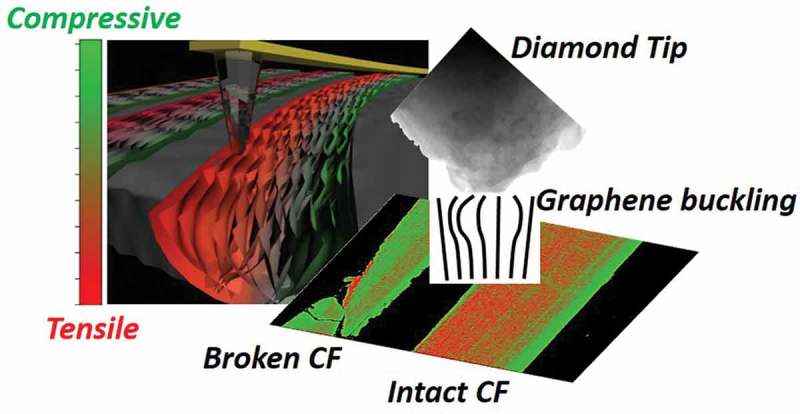

## 1. Introduction

Modulus characterization by atomic force microscopy (AFM) provides a new method of mapping residual stress in a material with nanometric resolution [1,2]. The operating principle can be described as: the lateral tensile/compressive stress preexisting in the specimen produces a partial force component that counters/enhances the vertically applied AFM probe force; such reduced/increased vertical force actually applied on the specimen would make the apparent Young’s modulus sensed by the AFM probe smaller/bigger than when no residual stress is presented; the distribution of such apparent Young’s modulus over a preassumed uniform material would thus reflect local stress distribution. This phenomenon of modulus hardening is similar to the superlattice modulus effect observed in metal films and modulus softening effect observed in nuclear graphite, but out of very different mechanism [3,4]. This technique is very attractive as nondestructive stress sensing for carbon fiber (CF)-based composite materials. Though widely used as filler materials for CF reinforced plastic (CFRP), the brittle nature of CF often causes catastrophic fatigue failure of the CFRP structural component [5,6]. It imposes danger for structural applications especially for transportation safety, because large passenger airplanes are now manufactured with more than half weight percent of CFRP based on polyacrylonitrile (PAN). The nanometric resolution provided by the new modulus mapping method thus could potentially resolve a single defect presented in the CF that may later develop into a fatal fracture [7]. Existing nondestructive stress characterization methods for CF, including surface roughness optical sensing [8], acoustic emission [9], infrared thermography [10], electric resistance [11], digital image correlation [12], Raman spectroscopy [13] and X-ray diffraction [14], do not offer a spatial resolution that is high enough for single defect detection.

Though the operation principle of the modulus mapping method is quite straight forward, one must have the preknowledge of CF Young’s modulus dependence on applied probe force in order to accurately correlate the modulus measurement results to residual stress values. This is because in the AFM-based modulus mapping, the force actually applied on the specimen is different with and without residual stress. The intrinsic influence from modulus change due to probe force difference, out of effect such as lattice anharmonicity, must be considered before extrinsic influence from residual stress can be successfully decomposed [15]. However, due to the high anisotropy of graphite atomic structure and the polygranular nature of most graphite-based material, the intrinsic dependence of elastic modulus on applied load force is quite complex. Large discrepancy exists in literature about both the elastic modulus values and their dependence on applied force. For example, Gauster and Fritz applied hydrostatic compressive pressure on a densified pyrolytic graphite and reported modulus values between 30 GPa and 1 TPa and relative modulus increases between 21%/GPa and 3%/GPa against stress increase [16]; Diss studied compressive modulus on pyrolytic graphite film and CF by applying stress through sharp tip indentation. The resulting moduli are all smaller than 100 GPa and could either increase or decrease with compressive force, which depends on the modulus of substrate underneath the measured location [17]. Yoda et al. applied Instron-type compressive force on isotropic graphite and found modulus value of 10 GPa with decreasing relative modulus with the rate of 642%/GPa [18]; Marrow et al. studied compressive surface of similar isotropic graphite with coarser grains under four-point bending load and found no change of elastic modulus with compressive force [19]. It is evident that the differences in the graphite-based material under study and the particular way of applying load force are the main causes for the scattered elastic modulus data in literatures. Reliable elastic modulus data must be investigated individually for a specific experimental setup.

In this study, we applied a pinpoint indentation method based on AFM to acquire modulus values on the surface of PAN-derived CF imbedded in a commercial CFRP. The modulus measurement is similar to those widely used in micro and nanoindentation methods, except that the maximum penetration depth in the pinpoint mode is only a few nanometers instead of the over-hundreds of nanometers depth used in nanoindentation and over-micrometer depth used in microindentation [17,20]. The pinpoint mode thus has much less influence from underneath substrate and could reflect only the mechanical property of the targeted location. It is therefore understandable that the pinpoint indentation modulus is much higher than those measured by uniaxial tensile test and deep indentation test, which are influenced by large-sized defects such as voids [21].

The main objective of this article is on the intrinsic modulus dependence on load force for PAN-derived CF, measured using the pinpoint indentation method. Intrinsic modulus changing mechanism was studied using an *in situ* AFM holder inside a transmission electron microscope (TEM), where atomic structure change in CF could be directly observed [22]. Focus was put on the buckling of CF graphitic layers, which phenomena were speculated many times in literature but have not been quantitatively investigated [23–27]. This microstructural information combined with numerical simulation gives a quantitative prediction of the indentation modulus response to externally applied stress. Finally, the established modulus–stress relationship was used to obtain local stress map on a CF subjected to tensile stress applied by an AFM *in situ* three-point bending holder. Stress concentration, defect formation, fracture and stress redistribution were directly characterized. The pinpoint indentation technique thus proves to be a highly sensitive and nondestructive method to monitor stress distribution and defect formation in CFRP components in operational conditions.

## 2. Experimental details

PAN-based high strength standard modulus CF (T700s) provided by Toray company was chosen for this study. The CFRP material used in this study is a unidirectional composite T700S/2592. It consists of the high strength CF T700S and an epoxy resin 2592 with the cure temperature of 130 °C. The unidirectional carbon/epoxy laminates were fabricated from the prepreg tape of P3252-20 (TORAY). They were laid up by hand and cured in an autoclave. The glass transition temperature of the epoxy resin in the laminates was about 100 °C. The CF is with nominal tensile modulus of 280 GPa and tensile strength of 2 GPa. A bar-shaped specimen with the dimensions of 30 mm × 1.5 mm × 1 mm was cut along the fiber direction and has its surfaces polished with alumina paste. This specimen was mounted onto a three-point bending holder, which could be mounted on the stage of an AFM (NX10, Park System) for pinpoint indentation experiment.

During pinpoint indentation, a sharp diamond tip with approximated cone geometry is scanned over the top surface of the CFRP specimen. At each pixel locations, the tip is pressed into the specimen surface until a preset indenting force is reached. Piezo stage records each indentation depth into the specimen, which was used to calculate the apparent elastic modulus at each pixel locations. What also been recorded is the stage height at each tip–specimen contact point and these height values could be used to generate a topological height map of the specimen. Three-point bending holder is used to apply a series of bending forces to the specimen during the pinpoint indentation experiment.

A thin slice of sample (3 × 3 × 0.1 μm) was dug out from the CF surface using focused ion beam processing. The slice was attached onto the tip of a tungsten needle which could be mounted onto an indentation holder for TEM (Nanofactory Instruments AB). The indenter tip of the holder can press into the CF slice with known force values, while microstructure of the CF could be observed by TEM. The TEM used in this study is a JEM-3100F operated at 300 kV.

The deformation of a CF during indentation was simulated using finite element method (FEM) implemented in a commercial software ABAQUS 6.14 [28]. The CF was assumed as a continuous body having different elastic properties in the in-plane direction and the stacking direction of graphene. The model consists of the fiber part and an indenter part as shown in Figure 3(b). The graphene layers are stacked in parallel with tilting *θ*° to the *yz*-plane in the fiber. Therefore, the *xy*-plane symmetrical model was generated, and its size was 58.6 × 24.3 × 24.3 nm. It has 15,358 nodes and 14,138 elements of 8-node hexahedron element with reduced integration (C3D8R). The mesh size was decreased in the region underneath the indenter, where the smallest mesh size was 0.3 nm. The elastic constants of graphite are cited from literature [29]. The sphere indenter of 10 nm in radius was generated as a rigid body assuming the stiffness of the diamond is higher enough than that of the carbon to ignore its deformation. The indentation was achieved by displacing the indenter by 0.2 nm in the *y*-direction.

## 3. Results and discussion

**Figure 1.**
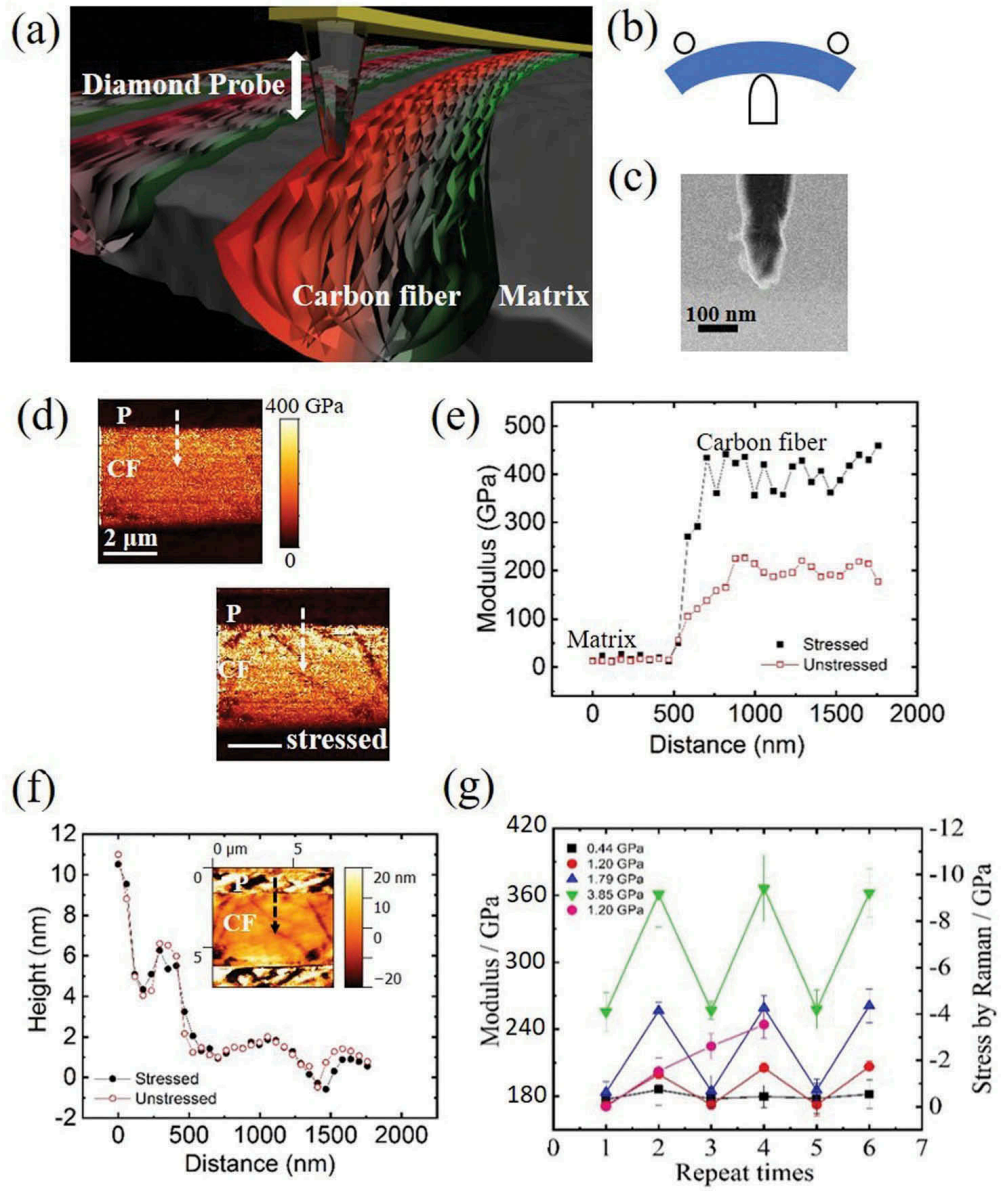
(a) Experimental principle showing the diamond tip indenting in the in-plane direction of graphene layers of a CF imbedded in polymer matrix; (b) schematics showing the three-point bending holder used to apply tensile stress to the CFRP specimen; (c) scanning helium ion microscopy image of the diamond tip after the experiment; (d) compressive modulus map of the same CF region with and without applied external tensile stress; (e) modulus line profiles created along the white arrows in (d) showing large modulus hardening of the stressed CF; (f) height line profiles created at the same positions as (e). Inset showing one height map of the same CF region; (g) indentation moduli obtained from a CF during cyclic tensile loading of four different average stresses with comparison to Raman spectroscopy measurements.

Figure 1(a) shows a schematic of AFM-based pinpoint indentation technique to characterize the local stress of the CF material. When a CF subjected to bending force, tensile and compressive stress is generated at outer and inner arch positions, which are represented by red and green color, respectively, an external stress was applied to CFRP bulk specimen by a three-point bending holder as shown in Figure 1(b). Each stress condition is named by the average stress applied on the observed region. The region near upper edge of CFRP under tensile stress is characterized by AFM. We used a diamond probe for stress measurement of CF. The helium ion microscopy image in Figure 1(c) shows that the radius of the diamond probe stayed around 15 nm even after 90 h of continuous measurement. Modulus values of each pixel were calculated automatically by AFM software using Hertz equation (Equation 2) by inputting maximum loaded force and tip indentation depth. The measurement of modulus could be affected by three factors: the inherent material property, surface morphology and external stress. Figure 1(d) shows the large change in the modulus of CF measured before and after the application of 3.85 GPa tensile stress. The modulus value is twice higher for the stressed CF than for stress-free CF. Modulus profiles before and after applying the external stress are created along the white arrows in Figure 1(d). The modulus profiles in Figure 1(e) showed that appreciable modulus change only occurs on CF, while that of *P* remains unchanged. We also took height profiles of the CFRP along the same region with modulus, as shown in Figure 1(f). The height profiles for CFRP before and after applying external stress exhibit the same value. An inset in Figure 1(f) shows such a height. It shows that tensile stress has no noticeable effect on the morphology of CFRP. Therefore, the changes observed in the modulus of CF before and after applying external stress are not caused by surface morphology of CFRP. Measured modulus response to four cyclic load–unload repetitions is plotted in Figure 1(g). The modulus values are obtained by averaging all pixels on CF region. Unstressed CF produces almost the same modulus except for those stressed to a high average value of 3.85 GPa. This is likely due to the viscoelastic nature of the epoxy matrix, which needs longer relaxation time after being stretched to high strain values or load cycled for many times. Since we compare only the relative change in modulus value *∆E/E*, the abnormal increase in unstressed modulus value would not introduce errors. In comparison, we also included stress values measured by Raman spectroscopy mapping. The red shift of 1590 cm^−1^ peak is averaged from 2D maps on about the same CF region during force–load cycles. We adopted the shift rate of 1.8 cm^−1^/GPa to calibrate the average stress values used in our experiments [30]. It is noted that Raman response deviated from expectation after the second cycle. This clearly suggests the sample heating effect due to the strong absorption of laser power for CF, even though we have chosen the lowest laser power that is still capable of detecting stress-related band shift.

An estimation was performed to understand whether the observed CF modulus increase is only the effect of surface membrane as observed in the case of Au film [1]. CF modulus is first assumed to be constant as probe loading force (*F_l_*) increased to 1000 nN full value. When the probe indents on the CF surface, the elastically deformed CF produces an elastic force *F_c_* that counters the probe to progress downwards. In the same time, the externally applied stress produced a vertical partial component force, *F_s_*, through surface membrane effect. The total loading force of the probe is equal to the sum of *F_s_* and *F_c_*, which follows the definitions of Refs. [1,31]:

(1) $$F_{s}=4\pi \cdot R\cdot d\cdot \sigma \cdot \cos\left[\tan^{-1}\left(2\sqrt{R/d}\right)\right]$$

(2) $$F_{c}=\frac{4}{3\left(1-\gamma^{2}\right)}E\cdot \sqrt{R}d^{1.5}$$

where *R* is the radius of diamond probe taken as 15 nm; *d* is the displacement of probe with respect to sample surface; *γ* is Poisson’s ratio taken to be 0.3. Taking the example presented in Figure 1(d): under a stressed condition with *σ* being 3.8 GPa, and the assumed constant modulus *E*_0_ being 200 GPa, *F_s_*+*F_c_ = F_l_ *= 1000 nN. We can calculate the corresponding *d* to be 0.75 nm by solving Equations (1) and (2). Since the apparent modulus *E* is determined by the AFM software assuming *F_c_ = F_l_ *= 1000 nN, Equation (2) is used again to obtain an apparent *E* value of 207 GPa. Therefore, there will be only 3.5% increase in *E* if surface membrane effect was the only cause. It is 30 times lower than the ~100% increase in *E* from the experimental observation as shown in the lower map in Figure 1(d).

**Figure 2.**
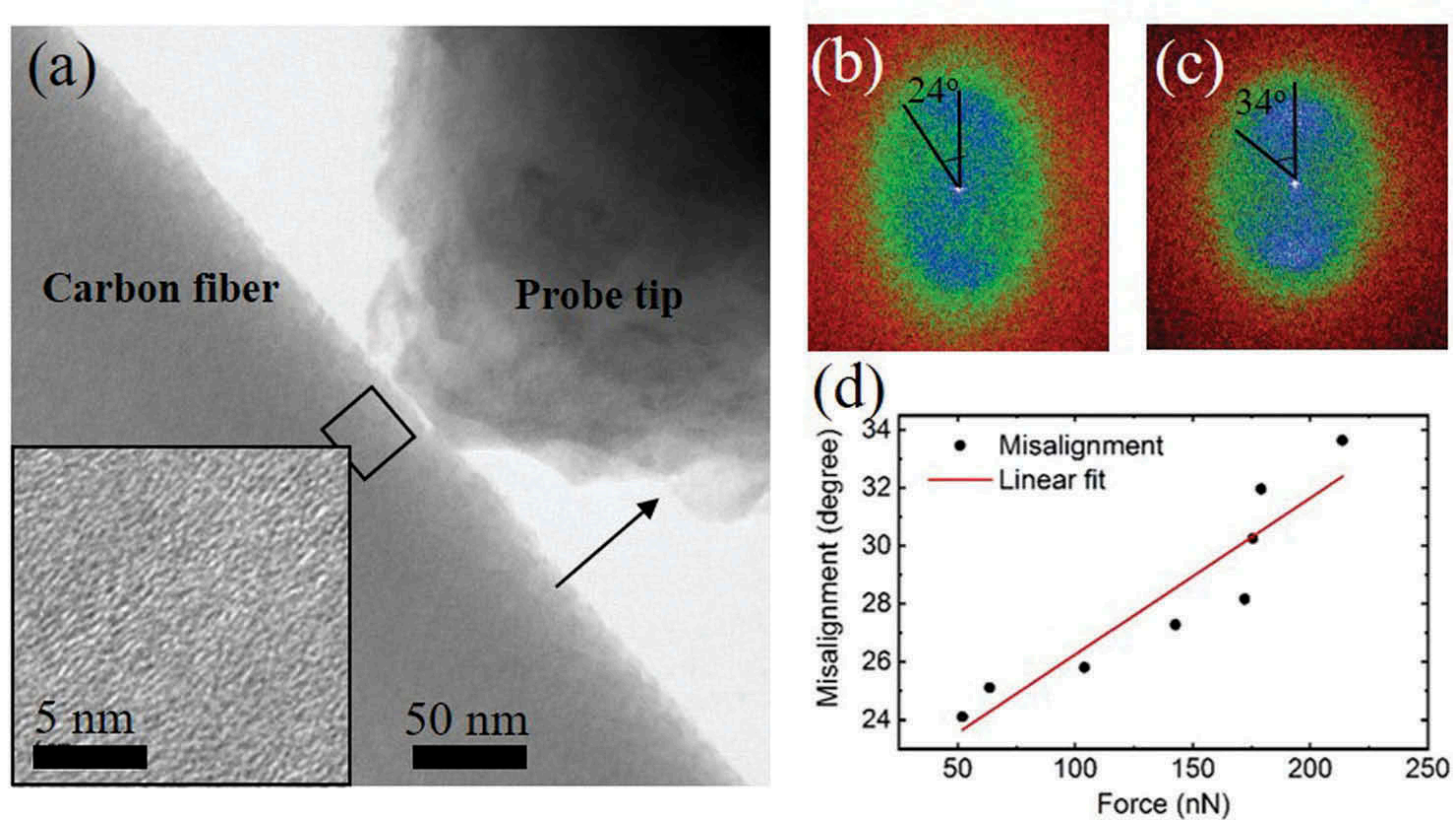
(a) Low magnification TEM image showing the AFM tip indenting on a specimen sliced from a CF. Inset shows high-resolution image about the indented region; (b,c) fast Fourier transform patterns of the same region as marked by the dark square in (a), where misalignment angle of graphene layers changed before and after the application of indenting force of 220 nN; (d) plot of misalignment angle with the applied indenting force to the CF specimen.

To understand the large indentation modulus hardening observed for CF with tensile stress, we have conducted an *in situ* study inside a TEM where an AFM probe indents a thin slice specimen cut from a CF. Low-magnification TEM showing the specimen and the AFM probe is presented in Figure 2(a) [22]. The AFM probe position is fixed, and the specimen moves in the dark arrow direction to perform indentation. The thickness of CF specimen is 100 nm. The inset in Figure 2(a) is a high-magnification image about a region marked by a dark rectangle near the edge of CF, where graphene layers are shown with misalignment to the in-graphene-plane axis. We performed fast Fourier transform (FFT) analysis on the high-resolution TEM images acquired from the same area as the AFM probe was pushed deeper into the CF specimen. FFT results of images taken before probe contact and on the deepest indentation are shown in Figure 2(b) and (c), respectively. FFT pattern presents an elliptical shape with long axis parallel to the in-graphene-plane axis. Perfectly aligned graphene layers would produce two spots symmetric about the pattern center. In the case of misaligned graphene layers, these two spots elongated into two arches with arch extension angles, which are used to define misalignment angles in this case. Such misalignment angle is plotted against the indentation force sensed by the AFM probe, as presented in Figure 2(d). The misalignment angle was found to increase with indentation force with roughly a linear dependence. The TEM observation is consistent with previous reports about compressive softening of graphite due to graphene layer buckling.

**Figure 3.**
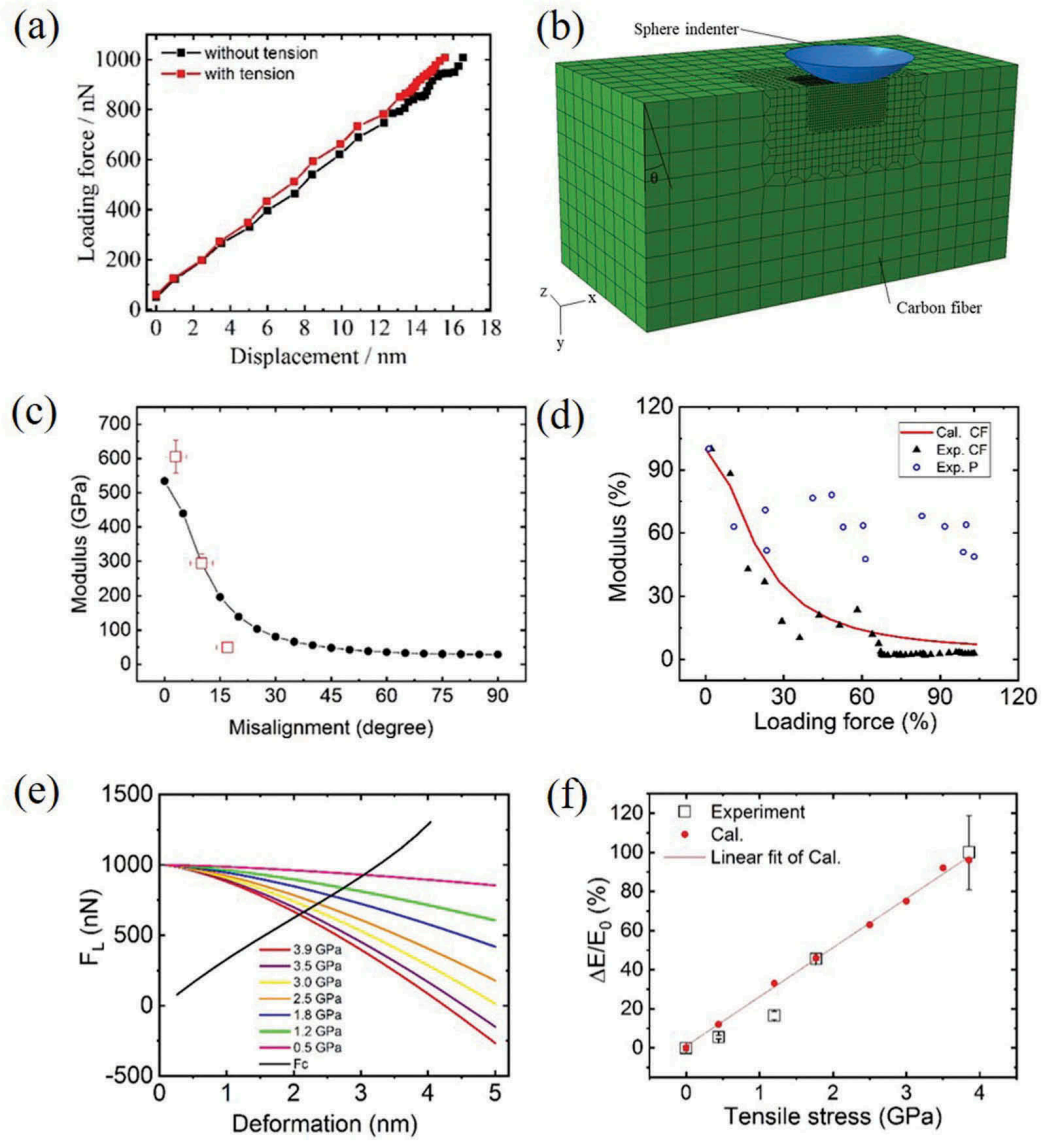
(a) Force–displacement curves obtained from the same position on a CF with and without external stress; (b) finite element method (FEM) model for indentation modulus simulation of carbon fiber; (c) simulation result of compressive moduli against misalignment angle for in-graphene-plane indentation; (d) calculated modulus of CF versus loading force compared with that of experimentally measured values and measured modulus response to loading force for polymer matrix; (e) plot of elastic force against deformation depth; dark line shows elastic force calculated from misalignment angle–force relationship obtained in the TEM experiment; colored lines show elastic forces expected by subtracting each stress forces from total load force; (f) plot of calculated modulus relative change against external tensile stress.

Figure 3(a) shows two typical force–displacement curves collected at the same location on CF with and without tensile stress during the modulus mapping shown in Figure 1(d). After subtracting deformation of the calibrated AFM cantilever, *d* was obtained for each *F_l_*. Using the Hertz Equation (2), we then obtained CF moduli *E* of 170 and 350 GPa with and without stress, respectively. This result is consistent with the 100% increase in *E* as calculated by software. To elucidate the effect of graphene layer misalignment on the measured indentation modulus, we have constructed a FEM model to simulate the modulus measurement using AFM probe. Graphene layer misalignment angle was simulated by tilting a perfect single crystalline graphite at an angle *θ* with respect to indentation axis, as shown in the illustration in Figure 3(b). Modulus was calculated following the definition in Equation (2) while *d* was simulated by FEM using model parameters as probe diameter being 15 nm and load force being 1000 nN. The simulation results of Figure 3(c) show that as the CF misalignment angle increases, the apparent modulus of CF decreases by as much as one order of magnitude [32]. The modulus drop is especially quick when misalignment angle is within the range between 0° and 45°, which corresponds to situation encountered during in-plane indentation in our setup. To verify the simulation result, we studied AFM indentation modulus obtained at planes of selected orientations from a block of highly oriented pyrolytic graphite (HOPG). HOPG was chosen as a model system for CF because its modulus value is similar to that of CF and both materials exhibit good elasticity even at small loading force, which is the scenario of this nanoindentation experiment. Bulk HOPG could be easily made into samples of defined graphene layer orientation (see supplementary information). Average HOPG moduli obtained at *θ* of 0°, 10°, 20° from HOPG indentation were also plotted in Figure 3(c). While the agreement was fair, it shows an even quicker drop of modulus with increased misalignment angle compared to simulation. It is because the experiment was carried out with same load force other than same indentation depth. Higher misalignment angle thus produces larger depth and results in a further reduction of apparent modulus. From Figures 2(d) and 3(c), the relationship between modulus *E* and loading force *F_l_* can be established through the misalignment angle after the correction of difference between AFM probe and TEM–AFM probe. Figure 3(d) is a comparison among such calculated *E*–*F_l_* curve for CF and experimentally measured data from CF and P. The loading force ranges for CF are chosen from 0 to 1000 nN while that for *P* is from 0 to 50 nN, because of their very different modulus values. It is shown that while measured modulus for CF shows a clear drop with increasing indentation force, agreeing quite well with calculation, that for *P* presents an almost constant modulus value. It proves our point that the modulus drop for CF is due to the strong heterogeneity of graphite structure while *P* is with an isotropic structure. According to the Hertz equation, we can thereby derive the relationship between the elastic force, *F_c_*, and the deformation depth, *d*, of CF, which is presented by a black curve in Figure 3(e). Under each deformation depth, the partial force component, *F_s_*, from externally applied stress can then be determined through Equation (1). In Figure 3(e), the cross points between the black line *F_c_* and the color lines (1000 nN-*F_s_*) give the deformations, *d_n_*, of CF under each external stress, *σ_n_*, during the same indentation load force of 1000 nN. The ratio of the deformation depth under stress, *d_n_*, over that without stress, *d*_0_, thus gives the relative increase of the apparent modulus, *∆E_n_*/*E*_0_, through Equation (3) as

(3) $$\frac{\Delta E_{n}}{E_{0}}=\frac{E_{n}}{E_{0}}-1=(\frac{d_{0}}{d_{n}}{)}^{1.5}-1$$

Finally, we arrive at the dependence of *∆E_n_*/*E*_0_ on external stress, *σ_n_*, which result is plotted in Figure 3(f). The consistency between the calculation and the experimental observation proved that indentation modulus softening must be considered to explain the large stress CF modulus response to orthogonal stress. In summary, externally applied stress caused only small change in AFM probe deformation depth; however, through the unique modulus softening of CF material, this small change in deformation causes much larger change in resistance force sensed by the indentation probe.

**Figure 4.**
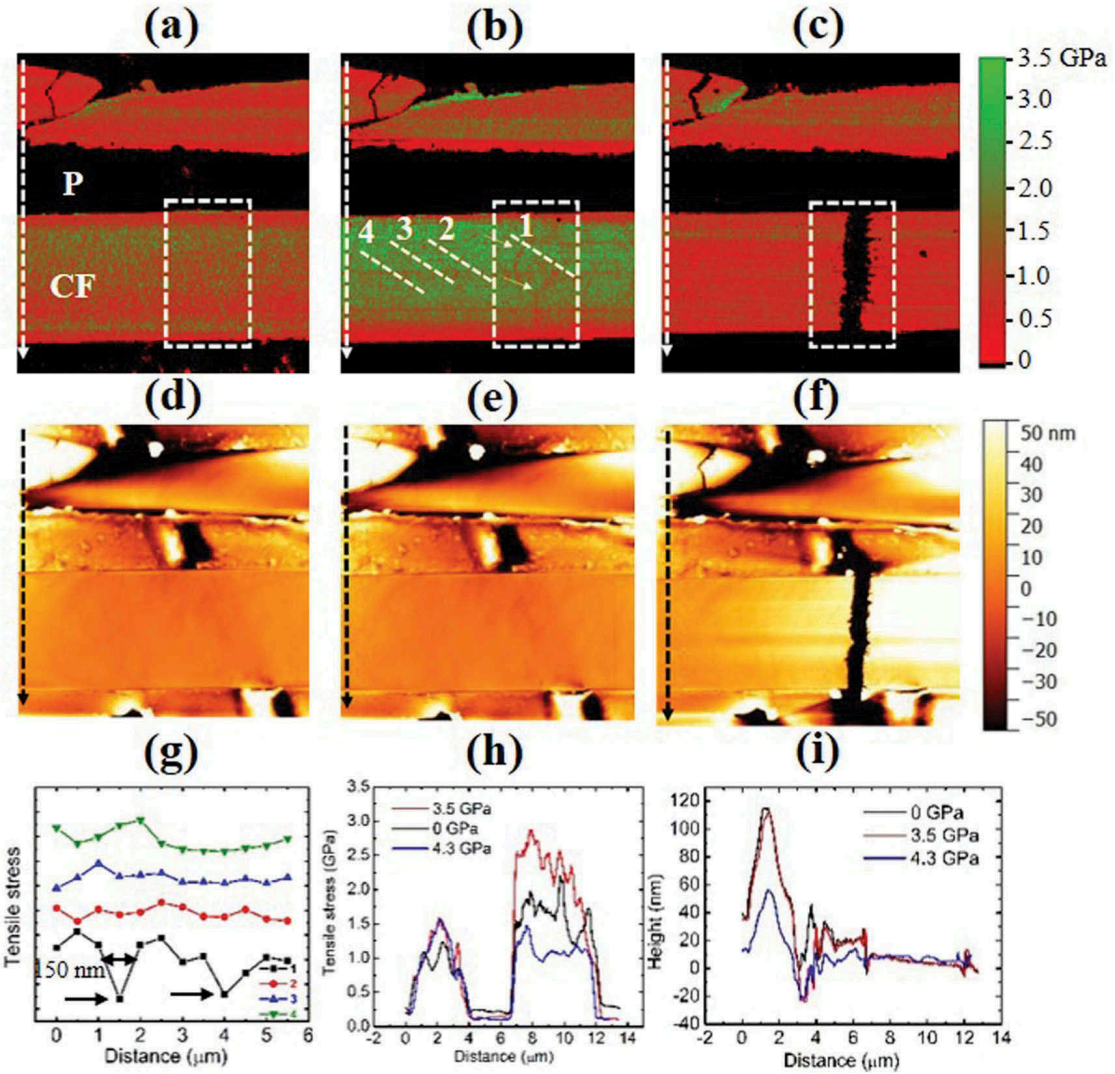
(a) Tensile stress map created in an area on CFRP containing a broken CF at the upper position and an intact CF at the lower position; (b) same region with applied external stress showing stress concentration on the lower intact CF and some newly formed defects; (c) fracture occurred at the lower CF when external tensile stress was further increased; (d–f) height map obtained simultaneously with modulus maps in (a–c); (g) modulus line profiles created at the position marked with 1–4 in (b), showing spatial resolution better than 150 nm at two defect locations; (h) modulus line profiles created along the white arrows in (a–c), respectively; (i) height line profiles created at the same position as in (h).

The above described modulus hardening of CF was utilized in the following example where stress distribution on CF was mapped by measuring local indentation moduli during an *in situ* fracturing experiment on a bulk CFRP material. As shown in Figure 4(a), upon applying a small tensile stress, the average stress of the lower intact CF is 2.0 GPa which is about 66% higher than 1.2 GPa of the upper broken CF. When specimen bending amount was increased, the lower CF average stress became 2.8 GPa. It is 86% higher than that of the upper CF, which only increased a little to 1.5 GPa. It is consistent with literatures that an ineffective length exists in broken CF over the range of which stress is no longer carried [33]. Two defect lines were also observed in the lower intact CF as marked by the two white arrows in Figure 4(b). Such defects were not presented in Figure 4(a) under a smaller stress. No difference in height maps between these two stress states could be noticed if one compares Figure 4(d) with (e). The defect lines exhibited lower modulus values compared to surrounding area. It suggests that local yield of material might have occurred, where local stress stopped increasing with increasing local strain. A line profile created crossing the defect is shown in Figure 4(g) as profile 1. The  full width at half maximum (FWHM) of the first defect line is 150 nm, which is consistent with previous observation about fracture-initiating defect size in CF [7]. Three other line profiles in area outside of the highlight were also created in Figure 4(b) to show that the defect contrast observed in profile 1 was not due to AFM noise. Next, the external stress was further increased till the lower CF fractured at the location of the previously observed defects. In Figure 4(c), the average stress on lower CF dropped to 1.1 GPa by 60%. The stress of the upper CF became 1.3 GPa, which is within 10% deviation of its initial state. This deviation should be experimental uncertainty. Figure 4(h) is a stress line profile of CFRP created along the white arrows in Figure 4(a–c). It is clearly seen that the stress in the upper CF did not change appreciably while that in the lower CF changed much more dramatically with the applied bending force to the CFRP specimen. Figure 4(i) is height line profiles taken along the black arrows in the height maps of Figure 4(d–f). The height profiles of the unstressed and 3.5 GPa stressed are identical, which agrees with our previous observation that the local modulus change did not come from the effect of the surface topography. The 4.3 GPa stressed CF showed a different height profile from the other cases, which might indicate CF–P debonding at the interface during the lower CF fracture [34].

## 4. Conclusions

It has been found that pinpoint indentation modulus of CF increased with external tensile tress in the orthogonal direction. The phenomenon was investigated in a quantitative way using TEM *in situ* indentation experiment and FEM modeling. The mechanism was applied as a method of sensing stress distribution in a CFRP bulk material during an AFM *in situ* fracturing experiment. Tensile stress was observed developing in the CF when external load increased. Nanometer-sized defects were found forming in the CF. As stress keeps increasing, the defects were found developing into a complete fracture and stress disappeared in the broken CF. The introduced technique thus provides a nondestructive way to monitor stress status of CFRP material to evaluate its load-carrying ability and to prevent catastrophic fracture. It would be interesting as one future plan to apply the technique on new CF–polymer composite where CF was pre-grafted with functional groups [35].

The phenomenon of stress-sensitive indentation modulus has been observed on CFRP in this study and reported on Au films through a similar AFM-based technique. In our recent experiment with indentation pre-stressed Si wafer, the modulus pattern mapped around the indentation pit also shows good agreement with stress field distribution confirmed by Raman microscopy (see supplementary materials). A general mechanism may exist for these observations on different material systems, which however first needs further investigation on each different case.
