# Supplementary material for: Stress dependence of indentation modulus for carbon fiber in polymer composite
Source: Sci Technol Adv Mater. 2019 Apr 26;20(1):412–20. doi: 10.1080/14686996.2019.1600202 (PMC6493271; doi:10.1080/14686996.2019.1600202)
Supplement: Supplemental Material [file TSTA_A_1600202_SM1848.docx]

Stress dependence of Indentation Modulus for Carbon Fiber in Polymer Composite Hongxin Wang^1^, Han Zhang^1^*, Daiming Tang^1^, Kenta Goto^1^, Ikumu Watanabe^1^, Hideaki Kitazawa^1^, Masamichi Kawai^2^, Hiroaki Mamiya^1^, Daisuke Fujita^1^*

^1^ National Institute for Materials Science, Sengen 1-2-1, Tsukuba, Ibaraki, 3050047, Japan. ^2^ Systems and Information Engineering, University of Tsukuba, Tsukuba, Ibaraki 305-8577, Japan.

*E-mail: ZHANG.han@nims.go.jp; FUJITA.daisuke@nims.go.jp

Supplementary Information

1. Indentation modulus on graphite planes of different graphene orientations

We chose a plate of highly oriented pyrolytic graphite (HOPG) (NT-MDT Co.) to use as our model system to investigate AFM indentation modulus dependence on graphene layer orientations. The plate was first cut into 3 plates about 4mmX4mmX1mm in size and glued on the sides of 3 Al blocks using hot wax. The sides of the corresponding Al blocks were pre-cut to form angles of 0^o^, 10^o^, 20^o^ with respect to top surface normal. The top surfaces were then sanded which was followed by multi-staged polishing with final finishing using SiO_2_ abrasive with 50nm particle size. The surface treatment process follows the same one with CFRP samples for better comparison basis. The HOGP surfaces with different graphene layer orientations were thus produced, as illustrated in figure S1 (a)-(c). The blocks were then investigated using AFM pinpoint mode with the same 1000nN load force with those for CF experiment. 128X128 pixels were indented over an area of 3umX3um for each block. The corresponding height maps ((d)-(f)) and modulus maps ((g)-(i)) are displayed in figure S1. Modulus mean values over the entire area with standard deviations were plotted in figure 3(c)


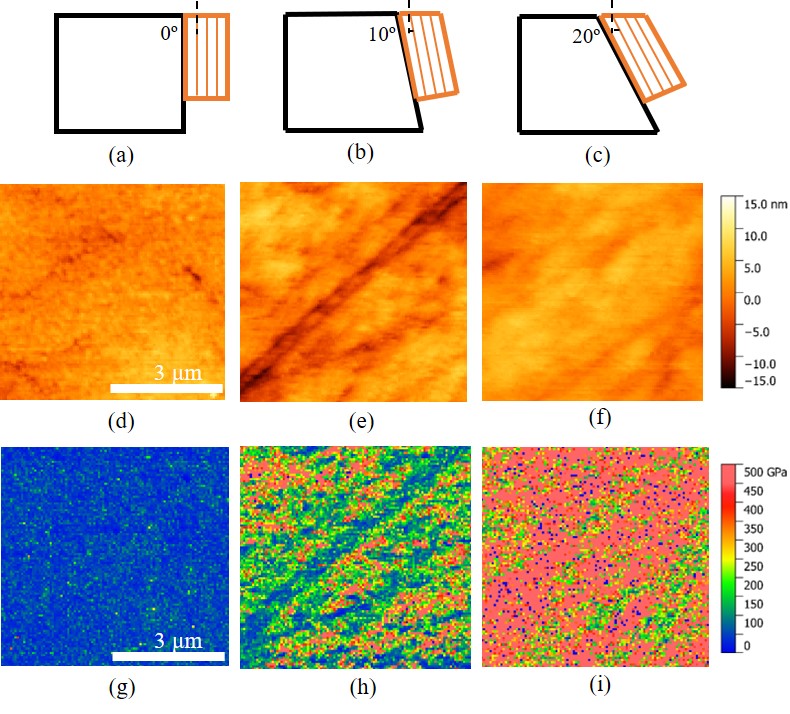


Figure S1. (a)-(c). Illustration showing the HOPG plates glued on Aluminum blocks with side surfaces forming different inclination angles with top surface normal; (d)-(f). Height maps created during AFM-based pinpoint indentation for HOPG samples in (a)-(c); (g)-(i). Modulus maps created simultaneously as (d)-(f).

1. Application for pre-stressed Si (100) characterization using a quantitative local stress characterization technique


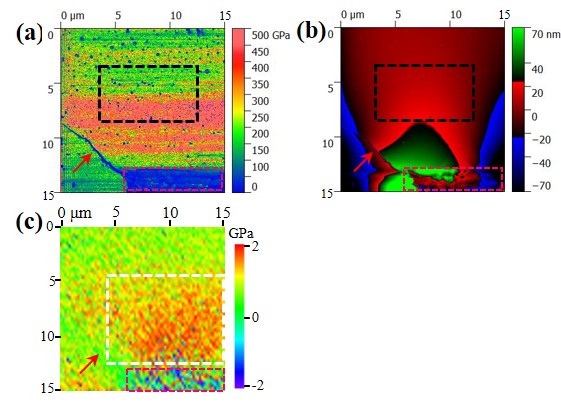


Figure S2. (a) Indentation modulus map created for pre-stressed silicon (100); (b) Height map obtained from pre-stressed Si simultaneously; (c) Raman map of the same sample region of Si.

As shown in figure S2, local modulus distribution and height map of pre-stressed silicon were obtained simultaneously using AFM-based pinpoint indentation technique. Modulus is a property that is inherent to materials. Local modulus can be affected by material state itself, such as surface morphology or induced external stress into the sample^1,2^. Figure S2 (b) shows height map of pre-stressed Silicon (100) wafer. There is height difference existing around the crack (red arrows) along indentation mark (pink rectangle) induced by a process in which an indenter engaged in and withdrew from the sample surface. The height value around the top region of the indentation mark is high while the modulus value in the same region is low. The part that is in the middle (black rectangle) of Si surface is flat with little height difference. However, the modulus that is obtained in the middle (black rectangle) of Si presents a changing distribution. This inconsistency proves that the detected modulus is affected by induced external stress.

A peak position of intrinsic Si is 520 cm^-1^ in Raman spectrum. The bond between Si atoms will be compressed or stretched when external stress is induced into Si. In this case, a peak shift of the collected Raman scattering is caused by the strained bond. Peak shifted to higher wavenumber in Raman spectrum causes the formation of the compressive stress while peak shifted to lower wavenumber causes the formation of the tensile stress. They are also called red shift or blue shift of the collected Raman scattering for compressive or tensile stress, respectively.^3^ From Raman map (c) of pre-stressed Si, it shows that stress inside the white square is compressive according to red shift of the collected Raman scattering. There is no peak shift around the crack. It proves that stress is released by the crack formation.

Comparing stress detection using AFM-based modulus characterization technique (a) to Raman mapping (c), the high modulus distribution in figure S2(a) is consistent with the compressive stress distribution in Raman map. The lower modulus distribution shows the intrinsic properties of Si wafer. Because no peak shift of the Ramen scattering stands for no stress induced into Si as shown in the green region of Raman map. Thus, it is confirmed that our technique can be used for Si material in stress characterization.

References

1. Polop. C; Vasco. E; Perrino. A.P; Garcia. R. *Nanoscale* **2017**, 9, 13938-13946, DOI: 10.1039/C7NR00800G

2. Gu. Y.Z; Li. M; Wang. J; Zhang Z.G. *Carbon* **2010**, 48, 3229-3235, DOI: 10.1016/j.carbon.2010.05.008.

3. Wang. H; Zhang. H; Da. B; Shiga. M; Kitazawa. H; Fujita. D. *Journal of Physical Chemistry C* **2018**, 122, 7187-7193, DOI: 10.1021/acs.jpcc.7b12415.
